# Transcutaneous Electrical Acustimulation Improves Gastroparesis Symptoms and Ameliorates Gastric Pace‐Making Activity in Patients With Diabetic Gastroparesis

**DOI:** 10.1111/nmo.70184

**Published:** 2025-10-22

**Authors:** Ying Zhu, Irene Sarosiek, Yan Sun, Jieyun Yin, Thomas Abell, Borko Nojkov, Richard McCallum, Jiande D. Z. Chen

**Affiliations:** ^1^ Division of Gastroenterology and Hepatology University of Michigan School of Medicine Ann Arbor Michigan USA; ^2^ Division of Gastroenterology Texas Tech University Health Science Center El Paso Texas USA; ^3^ Department of Internal Medicine Metrohealth Medical Center Cleveland Ohio USA; ^4^ Transtimulation Research Inc Oklahoma City Oklahoma USA; ^5^ Division of Gastroenterology, Hepatology and Nutrition University of Louisville Louisville Kentucky USA

**Keywords:** autonomic function, electrical stimulation, electroacupuncture, gastric slow waves, gastrointestinal motility, gastroparesis, neuromodulation

## Abstract

**Background:**

Gastroparesis is common in patients with diabetes. However, treatment options for diabetic gastroparesis are limited. Transcutaneous electrical acustimulation (TEA), a noninvasive method of delivering electrical stimulation via surface electrodes placed at certain acupuncture points that are in the vicinity of peripheral nerves, has been reported to exert therapeutic effects in patients with gastroesophageal reflux, functional dyspepsia, and constipation. The aim of this study was to explore the therapeutic potential of TEA for diabetic gastroparesis.

**Methods:**

Eighteen patients with diabetes were enrolled in a single‐center, placebo‐controlled, randomized and crossover trial with TEA and sham‐TEA. TEA was performed twice daily after lunch and dinner via acupoints, ST36 (below the kneecap in the vicinity of the peroneal, sciatic, and tibial nerves) and PC6 (wrist above the median nerve) for 4 weeks. A set of parameters previously reported to improve gastrointestinal motility was used for both TEA and sham‐TEA (delivered via sham‐points). The gastroparesis cardinal symptom index was used to assess symptoms. The electrogastrogram (EGG) was used to assess gastric pace‐making activity (slow waves).

**Key Results:**

The TEA treatment improved each of the five major gastroparesis symptoms in comparison to baseline: nausea reduced by 29.7% (*p* = 0.005), retching by 31.1% (*p* = 0.006), vomiting by 39.3% (*p* = 0.005), abdominal fullness by 21.4% (*p* = 0.005), and bloating by 20.6% (*p* = 0.006). There was also a significant improvement in the “pain interfering with activity” score with the 4‐week TEA treatment in comparison to baseline (*p* = 0.046). TEA improved gastric pace‐making activity, reflected as a significant increase in the percentage of normal gastric slow waves in the postprandial state (69.5% ± 12.1% vs. 77.4% ± 16.5%, *p* = 0.039). Concurrently, TEA resulted in a trend of postprandial increase in vagal activity.

**Conclusions and Inferences:**

TEA at acupuncture points ST36 and PC6 with appropriate parameters is effective in treating the major gastrointestinal symptoms in patients with diabetic gastroparesis. Further pivotal studies are warranted to determine its clinical efficacies.

## Graphical abstract

Gastroparesis is common in patients with diabetes. However, treatment options for diabetic gastroparesis are limited. In our study, transcutaneous electrical acustimulation, a noninvasive method of delivering electrical stimulation via surface electrodes placed at certain acupuncture points with appropriate parameters was effective in treating the major gastrointestinal symptoms in patients with diabetic gastroparesis.

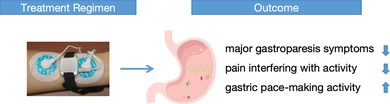

**Summary**

- Gastroparesis is common in patients with dibetes with limited treatment options.
- Transcutaneous electrical acustimulation (TEA) via a wearable medical device is a noninvasive method of neuromodulation.
- TEA at special acupoints with a set of special stimulation parameters improves major symptoms of gastroparesis in patients with diabetic gastroparesis.

## 1 Introduction

Gastroparesis is a disorder defined by objectively delayed gastric emptying and the presence of typical symptoms, such as early satiety, postprandial fullness, nausea and/or vomiting [1, 2]. Any condition that affects the neuromuscular function of the gastrointestinal (GI) tract can cause gastroparesis. It is a particularly common complication of diabetes mellitus and abdominal or thoracic surgeries [3, 4]. Gastroparesis is a common disorder as it affects up to 25%–30% of patients with functional dyspepsia (FD) and 40% of patients with type 1 diabetes [1, 3]. It significantly impacts the quality of life, increases direct healthcare costs, and is associated with high morbidity in affected patients.

Pathophysiologically, main mechanisms of gastroparesis include impaired gastric motility (gastric dysrhythmia, antral hypomotility and delayed gastric emptying), altered fundic relaxation or accommodation, and visceral hypersensitivity [5]. The multifactorial pathophysiology of gastroparesis likely contributes to the overall poor effectiveness of the available therapies in treated patients. For example, fundus‐relaxing medications have been studied for treatment of impaired accommodation, but these remedies may further delay the gastric emptying and increase the risk of associated vascular side effects [6]. Prokinetic agents are used to treat delayed gastric emptying but they can worsen the symptoms related to gastric accommodation because available prokinetics often impair the gastric accommodation [7]. Another therapeutic challenge in patients with gastroparesis is the treatment of abdominal pain that is present in over 50% of patients [3]. Unfortunately, gastroparesis patients are commonly prescribed with opioids for treatment of abdominal pain that worsens their gastrointestinal dysmotility and symptoms [8]. Therefore, an integrative method of treatment, able to address each of the pathophysiologic mechanisms of gastroparesis, is necessary.

The pathogenesis and underlying mechanisms of GI dysmotility in diabetes have not been completely elucidated. These factors include hyperglycemia, autonomic or enteric neuropathy, and loss of interstitial cells of Cajal (ICC) [9, 10]. The putative association of GI symptoms with disordered GI function arising from irreversible autonomic (vagal) neuropathy is long‐standing [11]. The vagus nerve plays a key role in gastric emptying and small intestinal motility. The vagal afferent fibers relay information to the nucleus of the solitary tract (brainstem). In turn, descending signals are conveyed by the vagal efferent fibers via the dorsal motor nucleus of the vagus, allowing for the so‐called vago–vagal reflexes [12]. Diabetic neuropathy is considered an important causal factor in gastric motility disorders of diabetes mellitus.

Electroacupuncture (EA) is an intervention that evolved from the conventional manual acupuncture where a weak electrical current is delivered to needles inserted into acupuncture points. Transcutaneous electrical acustimulation (TEA) is a needleless alternative to EA in which weak electrical stimulation is delivered via surface electrodes placed at acupuncture points. It has several distinctive features when compared to EA or traditional acupuncture [13, 14]: (1) TEA can be applied at acupuncture points that are in the vicinity of the peripheral nerves; (2) It uses stimulation parameters that can be optimized to affect the specific pathophysiologic mechanisms of gastroparesis and improve the sympatho‐vagal balance; (3) The stimulation can be applied daily (once or multiple times/day) as it is non‐invasive; (4) It can be applied via a discrete wearable device that is easily applicable and socially acceptable to patients [13, 15]. The most commonly used acupuncture points for the treatment of gastrointestinal (GI) symptoms are ST36 (a point below the kneecap in the vicinity of peroneal, tibial, and sciatic nerves) and PC6 (a point in the wrist coinciding with the median nerve) [15]. Previous studies have demonstrated that TEA with appropriate stimulation parameters improves gastrointestinal dysrhythmia, enhances gastric accommodation, and reduces visceral and abdominal pain in a variety of functional gastrointestinal disorders, including gastroesophageal reflux disease, functional dyspepsia, irritable bowel syndrome, and chronic constipation. However, it is unknown whether TEA exerts these effects in patients with diabetic gastroparesis.

This pilot study was designed to investigate the effects of TEA on upper gastrointestinal symptoms and gastric physiologic mechanisms, including gastric myoelectrical activity and autonomic functions in patients with diabetic gastroparesis, a subset with limited therapeutic options and distinct etiological mechanisms such as autonomic and peripheral neuropathy.

## 2 Methods

### 2.1 Study Participants

The study was performed in the Division of Gastroenterology at Texas Tech University Health Science Center, El Paso, Texas. Ten healthy controls (HC) and eighteen diabetic gastroparesis (DGP) patients with documented gastric emptying delay on standard scintigraphic study were prospectively enrolled in this randomized, single‐blind, crossover design study. The study was approved by the Institutional Review Board of the Texas Tech University Health Science Center.

Patient inclusion criteria were: (1) Males and females, 18–75 years old who were diagnosed with DGP at least 6 months prior to recruitment; (2) Presence of at least 1 severe gastroparetic symptom or 2 moderate gastroparetic symptoms (see assessment of gastroparetic symptoms below); (3) Delayed gastric emptying time (> 10% residual signal for solid content at 4 h after ingestion of the study meal) on a standard 4‐h gastric radionuclide scan [16] that had to be completed within the past 2 years; (4) Upper endoscopy or radiographic upper GI series study completed within the past year without evidence of obstruction, peptic ulcer, or any other structural finding to explain symptoms. Exclusion criteria were: (1) Uncontrolled diabetes with serum glucose levels predominantly > 300 mg/dL; (2) Any prior gastric surgery; (3) Advanced renal insufficiency; (4) Active malignancy; (5) Use of medications with known effects on gastric motility, such as prokinetics, anticholinergics, or opioids; (6) Pregnancy. Included healthy controls were also 18–75 years old, who had no history of GI disease, abdominal surgery, or systemic illness.

### 2.2 Randomization and Blinding

This was a single‐blind, randomized, sham‐controlled, crossover design trial. Patients were blinded to treatment modality and received a randomly assigned intervention, which was determined by the block randomization code. Once enrolled, patients were trained by one of the study team members for the placement of stimulation electrodes at the appropriate locations and the practical use of the stimulation device. The acupuncture points, ST36 and PC6, were not mentioned during the training, nor in the consent form. The clinical outcomes, including demographic data, symptom questionnaires, and physiological measurements, were collected and assessed by blinded investigators.

### 2.3 Study Protocol

**FIGURE 1:**
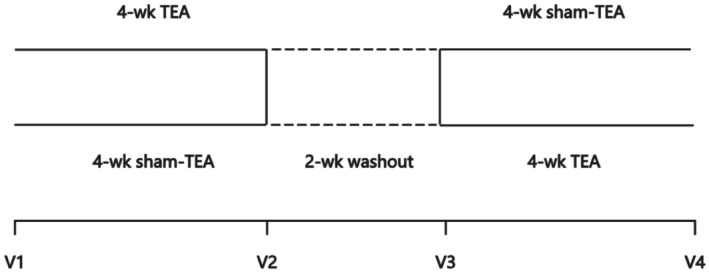
Experimental protocol. Sham‐TEA, sham transcutaneous electrical acustimulation; TEA, transcutaneous electrical acustimulation.

After one week of the phase‐in period, all qualified and enrolled subjects were randomly divided into two groups. One group was designated to cross over to 4‐week sham‐TEA after receiving 4 weeks of active TEA followed by a 2‐week washout. The second group was treated in a reversed order (see Figure 1). Patients were required to attend four clinical visits during the experiments. During each visit, patients underwent concurrent electrogastrogram (EGG, for assessing gastric pace‐making/slow wave activity) and electrocardiogram (ECG, for assessing the autonomic function) recordings.

### 2.4 TEA and Sham‐TEA

A watch‐size digital stimulator (SNM‐FDC01, Transtimulation Research Inc., Oklahoma City, OK) connected with the surface electrodes was used for TEA. The TEA treatment was given twice daily for 30 min before and 2 h immediately after lunch and dinner. Based on previous studies, TEA stimulation parameters were chosen as 2 s‐on, 3 s‐off, 25 Hz, 0.5 ms, and an amplitude of 2–10 mA, dependent on participants' tolerance (at a maximally comfortable and sensible level) [17, 18, 19, 20]. TEA was delivered via bilateral ST36 (Zusanli) on the legs and unilateral PC6 (Neiguan, either left or right hand at the choice of the subject) on the wrist (the reason for unilateral stimulation was to make the intervention more applicable for participants). Two digital stimulators were used for each patient, one for TEA at ST36 and the other for TEA at PC6.

**FIGURE 2:**
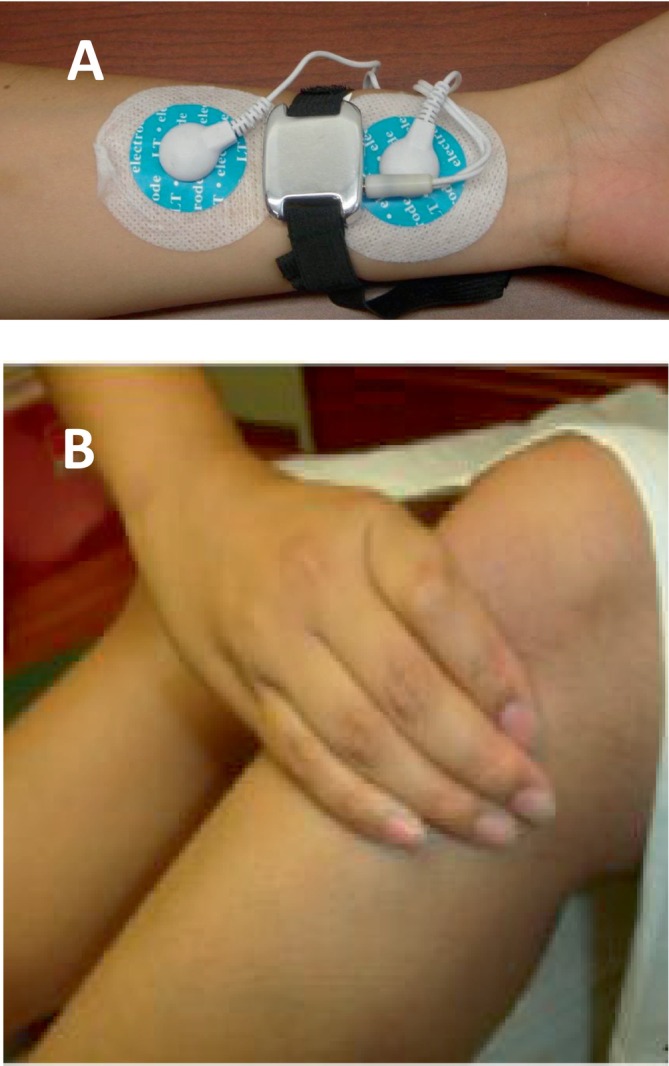
Placement of stimulation electrodes. (A) Placement of electrodes and TEA device at unilateral PC6. (B) Location of ST36. TEA was performed at bilateral ST36 points.

The acupoint ST36 was located four finger‐breadths distally from the outer eye of the knee between the fibula and tibia, one finger‐breadth measurement beside the tibia (Figure 2A). For TEA at PC6, one electrode was placed at PC6, located at one‐sixth of the remote end and five‐sixths of the proximal end of the stripe connecting the transverse wrist crease and cubital crease. The other PC6 electrode was positioned 4 cm above, along the same meridian (Figure 2B).

Sham‐points for bilateral ST36 were located 10–15 cm distal and 3–5 cm lateral from ST36 (not on any meridian). Sham‐points for PC6 were located 15–20 cm away from PC6 (up to the elbow and outside the coastal margin of the forearm, not on any meridian). The stimulation parameters for sham‐point stimulation were the same as the acupuncture point stimulation.

### 2.5 Assessment of Gastroparesis Symptoms

The Gastroparesis Cardinal Symptom Index (GCSI) was used to assess the severity of symptoms associated with gastroparesis based on three subscales: nausea/vomiting, post‐prandial fullness/early satiety, and bloating [21]. The nausea/vomiting subscale was comprised of the following three items: nausea, retching, and vomiting. The post‐prandial fullness/early satiety subscale was comprised of the following four items: stomach fullness, inability to finish a normal‐sized meal, feeling excessively full after meals, and loss of appetite. The bloating subscale was comprised of the following two items: bloating and stomach or belly visibly larger. The GCSI was assessed before and after the treatment at V1 (baseline), V2 (after first phase treatment), V3 (after 2‐week washout), and V4 (after the second phase treatment) (Figure 1). Each symptom was graded from 0 to 5 (0: none, 1: very mild; 2: mild; 3: moderate; 4: severe, and 5: very severe), with higher scores reflecting greater symptom severity. The GCSI total score was constructed as the average of the three symptom subscales.

### 2.6 Assessment of Health‐Related Quality of Life

RAND‐36 is a 36‐item questionnaire which generates eight health‐related quality of life domains: physical functioning (PF), role limitations due to physical problems (RP), bodily pain (BP), general health perception (GH), vitality (VT), social functioning (SF), role limitations due to emotional problems (RE), and mental health (MH). The questionnaire was assessed at V1 (baseline), V2 (after the first treatment phase), V3 (after a 2‐week washout, using as baseline for the second treatment), and V4 (after the second treatment phase) (Figure 1). Each dimension had a score range from 0 to 100; the higher the score, the better the quality of life [22].

### 2.7 Assessment of Gastric Pace‐Making Activity by Electrogastrogram (EGG)

A four‐channel electrogastrogram (EGG) device (Medtronic Inc., Minneapolis, MN) was used to record gastric slow waves noninvasively using previously established methods [23, 24]. Patients were in a supine position and asked not to talk or read during the test. Firstly, the abdominal skin was cleaned with a special gel (Nuprep, Weaver and Company, Aurora); then conductive gel (Ten20, Weaver and Company, Aurora) was applied to reduce the skin‐electrode impedance; lastly, six cutaneous electrodes were placed on the abdominal skin surface (a reference electrode: at the xiphoid process, a grounding electrode: the lower edge of the left rib arch, electrode 3: at the midpoint of the xiphoid and the umbilicus, electrode 4: 4–6 cm on the right of the horizontal of electrode 3, electrode 2: 5 cm to the upper left of electrode 3, and electrode 1: 5 cm to the upper left of electrode 2). The four‐channel EGG signals were recorded during 4 visits for 1 h in the fasting state and 1 h after a small test meal (230 Calories with 27 g of egg components and 6 saltine crackers) suitable for patients with gastroparesis [25].

The validated spectrum analysis method was used to obtain the following parameters [26, 27]: (1) percentage of normal slow waves [2.0–4.0 cycles/min (cpm)], representing the regularity of gastric slow waves; and (2) dominant power (DP) and dominant frequency (DF), representing the amplitude and frequency of gastric slow waves, respectively; (3) The percentage of coupling between two channels of gastric slow waves was calculated on a minute‐by‐minute basis. The minute was defined as coupled if the difference in the dominant frequencies of the two channels was equal to or smaller than 0.2 cpm. Otherwise, the minute was defined as uncoupled. The average percentage of coupling was defined as the mean value of all exhaustive pairs of channels. This parameter was used to assess the coordination/synchronization of gastric slow waves among different locations. A small test meal with 230 Cal was used to assess the postprandial changes in gastric pace‐making activity and autonomic function.

### 2.8 Assessment of Autonomic Function

Concurrent with the EGG recording, ECG was recorded with the ECG amplifier (ECG‐201; Ningbo Maida Medical Device) via ECG cutaneous electrodes placed on the right manubrium of the sternum, the fifth interspace in the left medioclavicular line, and the right chest (the ground electrode), respectively [19]. A heart rate variability (HRV) signal was derived from the ECG by detection and interpolation of R‐R intervals. The autonomic nervous function was assessed by spectral analysis of HRV, utilizing a previously validated method. The following spectral analysis HRV outcomes were derived: low‐frequency (LF, 0.05–0.15 Hz) known to reflect mainly sympathetic activity, high‐frequency (HF, 0.15–0.5 Hz) reflecting vagal activity, and LF/HF reflecting the sympathovagal balance [28].

### 2.9 Statistical Analysis

All analyses were performed using SPSS 23.0 software. The Shapiro–Wilk test was used to assess data distribution. Descriptive statistics for quantitative variables are presented as means ± SD. In this crossover study, we had two baseline recordings, one at the beginning of the first treatment phase and the other at the beginning of the second treatment phase after crossing over and the washout period. All outcome measurements after the TEA or sham‐TEA treatment were compared with the corresponding baseline values, that is, the first baseline for the first phase of the treatment and the second baseline for the second phase of treatment. Analysis of variance (ANOVA) was used to compare the difference among different groups or different recording channels. Chi‐square analysis was performed to study the categorical variables. Statistical significance was set at *p* < 0.05.

## 3 Results

### 3.1 Demographic Characteristics of Study Participants

Twenty‐six patients with DGP were consecutively enrolled and 18 patients completed the study (mean age: 50.4 ± 10.8 years, 13 females). Ten healthy individuals (mean age: 44.2 ± 13.3 years, 7 females) were also included in this study for baseline EGG and ECG recording without TEA or sham‐TEA treatment. The demographic characteristics were matched between the two groups, including age, gender, height, and weight (Table 1).

**TABLE 1:** Characteristics of DGP patients and healthy controls.

	DGP (*n* = 18)	HC (*n* = 10)	*p*
Age (year)	50.4 ± 10.8	44.2 ± 13.3	0.916
Height (cm)	166.4 ± 11.9	165.7 ± 11.6	0.844
Weight (kg)	81.6 ± 18.8	78.6 ± 19.7	0.846
BMI (kg/cm^2^)	29.4 ± 8.0	28.7 ± 8.2	0.835
Gender (female %)	13 (72.2)	7 (70.0)	0.901
Ethnicity (*n* %)			0.062
Hispanic	12 (66.7%)	10 (100%)	
African American	6 (33.3%)	0 (0%)	

### 3.2 TEA Treatment Adherence

Good compliance with the TEA treatment was observed by study participants. The average usage of the therapy was 300.3 ± 82.5 min/day in the TEA group and 275.7 ± 65.9 min/day in the sham‐TEA group (requested usage: 300 min/day). This information was obtained from the TEA devices that automatically recorded each usage.

### 3.3 Effects of TEA on Gastroparesis Symptoms

**FIGURE 3:**
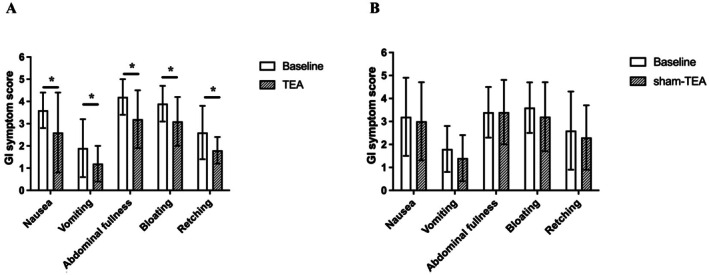
Effects of TEA on gastroparetic symptoms (*n* = 18). (A) TEA improved 5 of 9 Gastroparesis Cardinal Symptom Index (GCSI). (B) Sham‐TEA had no effect on gastroparetic symptoms. *p < 0.05 versus baseline.

The TEA treatment was effective in significantly improving each of the five major gastroparesis symptoms in comparison to baseline (Figure 3): nausea reduced by 29.7% (*p* = 0.005), vomiting by 39.3% (*p* = 0.005), abdominal fullness by 21.4% (*p* = 0.005), bloating by 20.6% (*p* = 0.006), and retching by 31.1% (*p* = 0.006). There was no significant difference in any of the symptom scores before and after sham‐TEA treatment.

### 3.4 Effects of TEA on Health‐Related Quality of Life

There was significant improvement in the “pain interfere activity” score with the 4‐week TEA treatment in comparison to baseline (3.7 ± 0.8 vs. 3.1 ± 1.4, *p* = 0.046), but there were no statistically significant changes in other quality of life dimension scores (*p* > 0.05) with the TEA. There were no statistically significant differences before and after sham‐TEA treatment in any of the quality of life dimension scores.

### 3.5 Effects of TEA on Gastric Pace‐Making Activity

#### 3.5.1 Percentage of Gastric Slow Waves

**FIGURE 4:**
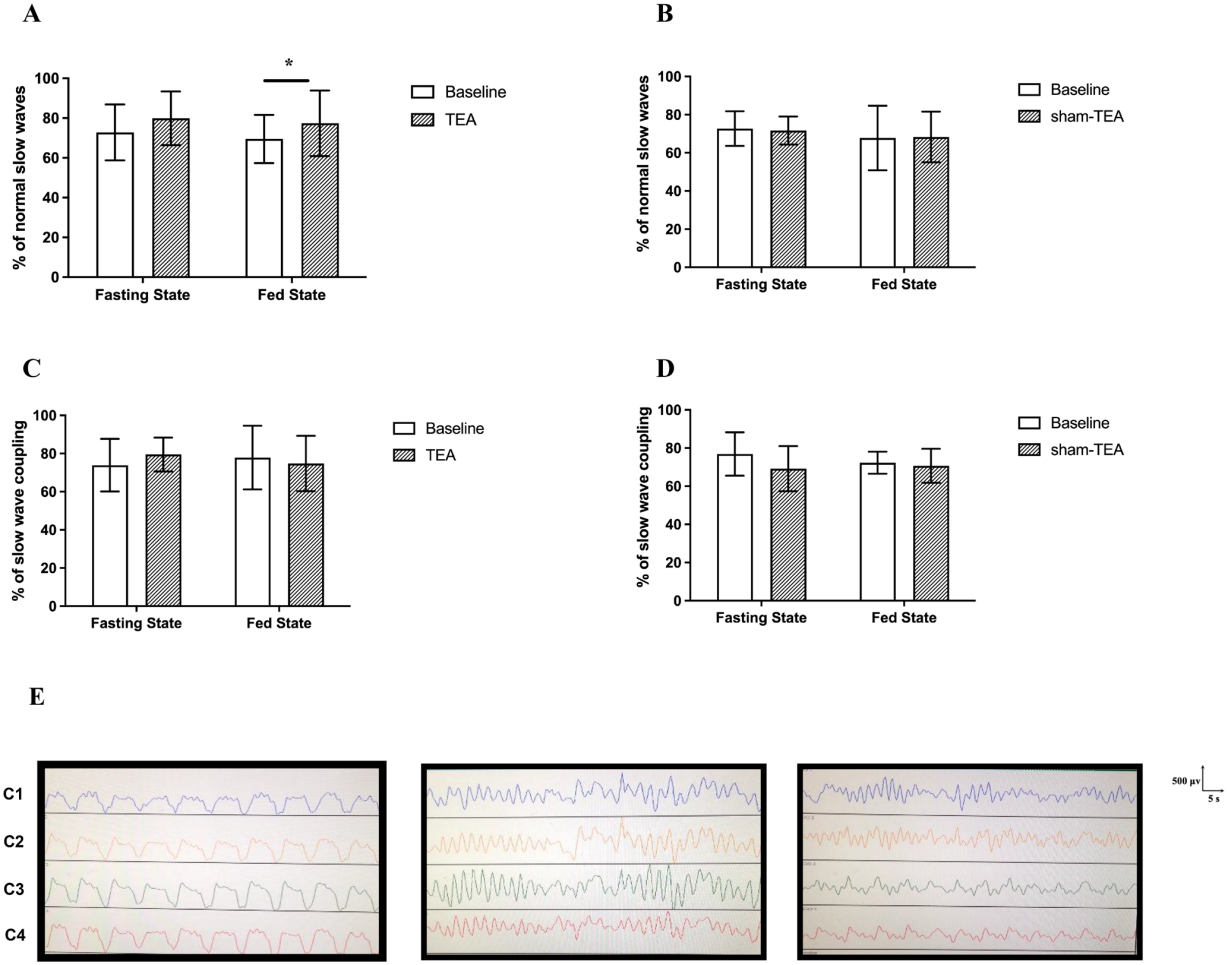
Effects of TEA on the percentage of gastric slow waves and coupling (*n* = 18). (A, B) Four‐week TEA, but not sham‐TEA, improved the percentage of normal slow waves (% NSW) in the fed state. (C, D) Four‐week TEA, but not sham‐TEA, showed a trend of increase in the percentage of normal slow wave coulping in the fasting state. * *p* < 0.05 versus baseline. (E) Typical EGG tracing in a healthy control (left), a patient before the treatment (middle) and the patient after the treatment (right).

The average percentage of normal gastric slow waves (% NSW) for the 4 recording channels in patients with DGP was significantly decreased compared to healthy controls in fasting (72.8% ± 14.1% vs. 82.7% ± 7.9%, *p* = 0.016) but not in postprandial states (69.5% ± 12.1% vs. 74.4% ± 5.2%, *p* = 0.052). Of the four channels, the % NSW in channel 1 showed the most significant decrease compared to healthy subjects (65.3% ± 19.3% vs. 79.6% ± 15.1% in the fasting state, *p* < 0.05; 61.1% ± 12.1% vs. 68.3% ± 11.5% in the postprandial state, *p* < 0.05). After 4‐week TEA treatment, the average % NSW of the four channels was significantly increased compared to the baseline not in fasting (72.8% ± 14.1% vs. 79.9% ± 13.5%, *p* = 0.053) but in postprandial states (69.5% ± 12.1% vs. 77.4% ± 16.5%, *p* = 0.039) (Figure 4A,B); TEA significantly improved the % NSW in channel 1 in both fasting (64.5% ± 16.3% vs. 75.1% ± 22.3%, *p* = 0.028) and postprandial state (61.2% ± 12.1% vs. 72.3% ± 17.1%, *p* < 0.01). Typical EGG tracings are presented in Figure 4E.

#### 3.5.2 Dominant Frequency and Power

The dominant frequency (DF) was not significantly different between the patients with DGP and healthy controls in both fasting (3.12 ± 0.35 vs. 2.91 ± 0.09, *p* > 0.05) and postprandial states (3.03 ± 0.55 vs. 3.11 ± 0.13, *p* > 0.05). Similarly, there was no significant difference in dominant power (DP) between these two groups. The TEA treatment did not lead to any significant changes in either DF or DP.

#### 3.5.3 Percentage of Slow Wave Coupling

The percentage of slow wave couplings among the 4 channels was slight but significantly reduced in DGP patients compared with healthy subjects (73.9 ± 13.8 vs. 78.9 ± 10.6, *p* < 0.05) in the fasting state but not in the postprandial state (73.3 ± 8.9 vs. 75.3 ± 6.4, *p* > 0.05). The 4‐week TEA resulted in a trend of increase in the coupling of gastric slow waves in the fasting state (73.9 ± 13.8 vs. 77.9 ± 16.7, *p* = 0.06) but not in the postprandial state (73.3 ± 8.9 vs. 74.8 ± 14.5, *p* > 0.05) (Figure 4C,D).

### 3.6 Possible Mechanisms of TEA Involving Autonomic Functions

**FIGURE 5:**
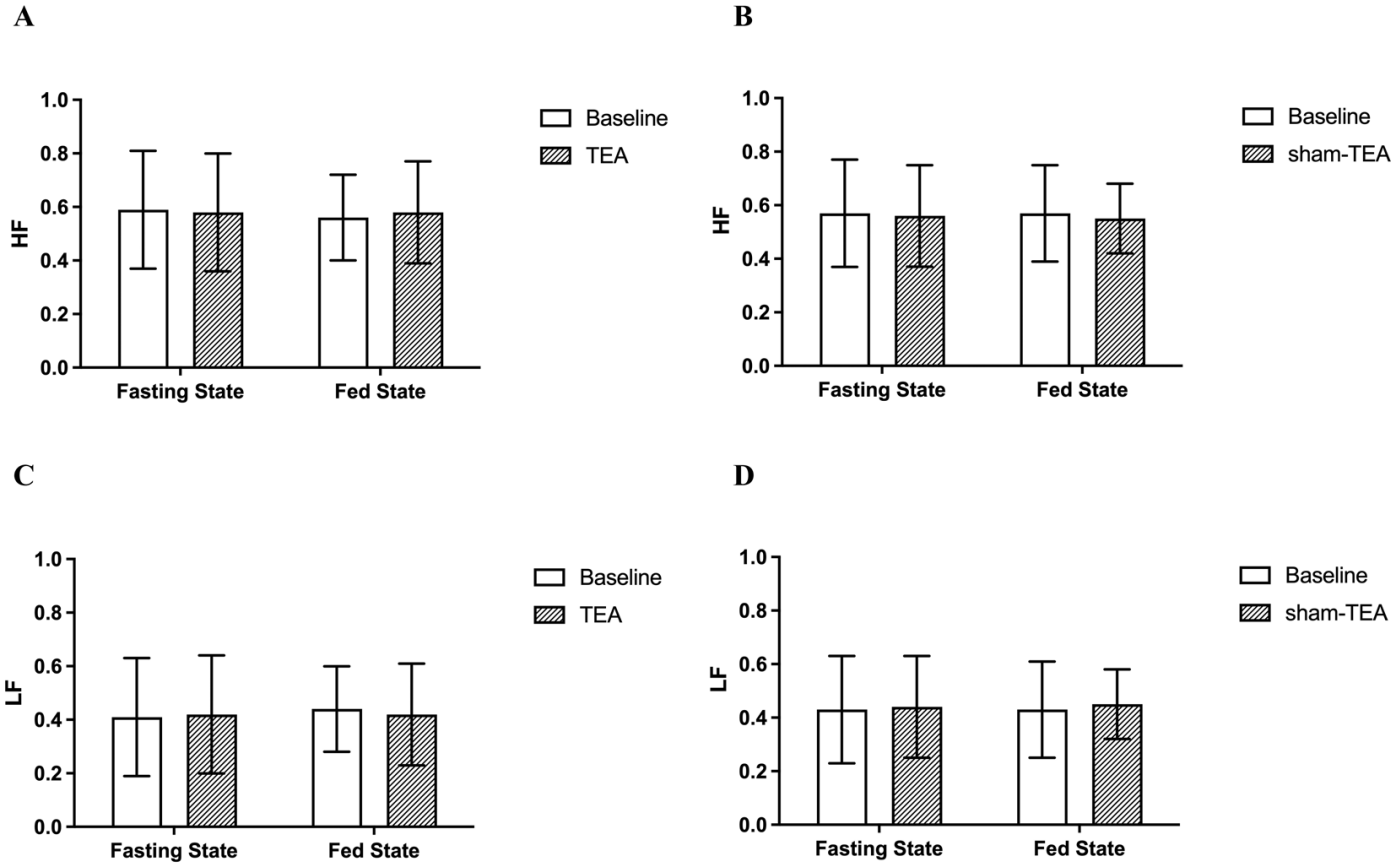
Effects of TEA on autonomic mechanisms (*n* = 18). (A, C) TEA showed a trend of increased HF (indicating vagal activity) and decreased LF (indicating sympathetic activity). (B, D) Sham‐TEA had no significant effect on either vagal or sympathetic activity. HF, high frequency; LF, low frequency.

The healthy volunteers showed a trend of significant postprandial change in LF (0.52 ± 0.16 vs. 0.58 ± 0.18, *p* = 0.086), HF (0.48 ± 0.16 vs. 0.42 ± 0.18, *p* = 0.086), and LF/HF (1.31 ± 0.7 vs. 1.81 ± 1.3, *p* = 0.078). However, this trend of changes was not noted in DGP patients (LF/HF: 0.83 ± 0.15 vs. 0.84 ± 0.15, *p* > 0.05; HF: 0.59 ± 0.22 vs. 0.56 ± 0.15, *p* > 0.05), suggesting an impaired autonomic response to the ingested food. Four‐week TEA, but not sham‐TEA, resulted in a trend of postprandial increase in vagal activity (HF) (0.56 ± 0.15 vs. 0.58 ± 0.19, *p* = 0.084) and postprandial decrease in sympathetic activity (LF) (0.44 ± 0.17 vs. 0.42 ± 0.18, *p* = 0.084) (Figure 5).

## 4 Discussion

In this study, we found that: (1) The 4‐week TEA but not sham‐TEA treatment reduced the symptom severity scores of the five major gastroparesis symptoms and the score of “pain interfere activity” regarding the quality of life. (2) Patients with DGP showed a reduced percentage of normal gastric slow waves and the percentage of slow wave couplings in comparison with healthy controls. The 4‐week TEA but not sham‐TEA increased the percentage of normal gastric slow waves and resulted in a trend of increased couplings of gastric slow waves; (3) These ameliorating effects were accompanied by a trend of postprandial increase in vagal activity (HF), suggesting a possible autonomic mechanism.

The patients enrolled in this study had severe symptoms of fullness, nausea, and bloating (mean score about 3–4) that might not be representative of all patients with DGP. Also, the patients had moderate/severe pain (mean score ~4). The majority of patients with severe symptoms could be attributed to two factors: (1) The hospital where the study was performed was a tertiary medical center with extensive experience in treating gastroparesis, and therefore, patients with more severe symptoms came to the hospital; and (2) the pharmacological treatment was reported to show limited effects, especially for pain. About 40% of patients reported using opioids, with abdominal pain being the indication in 60% of the patients. Patients on opioids had more gastric retention, worse quality of life scores, and more hospitalizations and use of antiemetic or pain modulator medications as compared with non‐users [29].

While TEA at acupoints ST36 and PC6 has been shown to improve gastric motility and symptoms in patients with GI motility disorders, such as gastroesophageal reflux, chronic nausea and vomiting, functional dyspepsia, and constipation [15, 20, 30, 31], it was unknown whether TEA had similar effects in patients with diabetic gastroparesis, a subset with limited therapeutic options and distinct etiological mechanisms such as autonomic and peripheral neuropathy. In the current study, we found that needleless TEA, utilizing stimulation parameters previously shown to improve gastric motility, substantially reduced the major gastroparesis symptoms in a cohort of patients with diabetic gastroparesis. This was an important preliminary finding as even though this was a small study, it was prospective, sham‐controlled, and randomized. Furthermore, the sham‐TEA treatment was performed with the same electrical stimulation but at non‐acupoints. The study showed substantial improvement, in the range of 30%–40%, of the most cumbersome symptoms of nausea and vomiting. Nausea is the most common symptom (more than 95% of patients) of gastroparesis and pharmacological treatment modalities frequently have limited effectiveness and associated side effects which limit their practical use. Therefore, it is of great clinical significance to further investigate the therapeutic effectiveness of the longer time use of TEA for DGP. Previously, transcutaneous cervical vagal nerve stimulation was reported to improve symptoms of gastroparesis in patients with refractory gastroparesis [32, 33]. In these studies, noninvasive electrical stimulation was performed using a hand‐held device; however, these were open‐label studies and possible placebo effects could not be ruled out.

In addition to the improvement of classic gastroparesis symptoms, TEA also showed an analgesic effect. In the present study, a significant improvement was noted in the pain‐interfering activity with TEA in comparison with Sham‐TEA. The TEA parameters in this study were not designed for treating pain, yet the treatment showed an ameliorating effect on pain assessed by the overall RAND‐36 interference score. Previously, a similar TEA intervention but with different parameters (100 Hz instead of 25 Hz) was reported to improve visceral pain in patients with irritable bowel syndrome and animal models of visceral hyperalgesia [34, 35]. The findings in the current and previous studies suggest that TEA with appropriate stimulation parameters might be a novel option to also treat abdominal pain in patients with DGP. It has been reported that EA inhibits pain through peripheral, spinal, and supraspinal pathways with the involvement of a series of bioactive molecules including opioid, serotonin, norepinephrine, and cytokines, among others [36, 37]. We speculate that, mechanistically, TEA might follow a similar pathway of EA to alleviate abdominal pain.

The EGG was shown to be a reliable and accurate noninvasive technique for the assessment of gastric pace‐making activity [27], which correlates positively with gastric motility and gastric emptying [38, 39]. Antra‐duodenal motility was not assessed in this study due to the invasive nature of the test; instead, we used the noninvasive EGG as a surrogate of gastric motility. In this study, the 4‐week TEA increased the percentage of normal gastric slow waves and resulted in a trend of increase in the couplings of gastric slow waves recorded from different channels/locations. The percentage of normal slow waves represents regulatory activity of the gastric pace‐making system. Slow wave coupling reflects spatiotemporal coordination of gastric slow waves. Improved slow wave regularity and coupling after the TEA treatment suggested enhanced gastric motility and spatial synchronization, supporting a mechanistic role of TEA. A number of previous studies reported that EA or TEA at ST36/PC6 using the same parameters improved gastric pace‐making activity and GI motility in animals [40, 41] and patients with gastric dysmotility [20, 42].

Given the role of the autonomic nervous system in the monitoring and regulation of gastrointestinal motility, we examined its function by using the power spectral analysis of heart rate variability, which is a noninvasive and simple method for the quantitative evaluation of autonomic function [28]. We found that postprandial autonomic function was impaired in patients compared to healthy controls, supporting a possible pathophysiological role for autonomic dysfunction in diabetic gastroparesis. There was a trend of improved autonomic function (postprandial increase in vagal and decrease in sympathetic activity) after the TEA treatment, although this did not reach statistical significance. The no significance might be attributed to the following factors: (1) a large intersubject variation in the autonomic function assessed by the spectral analysis of heart rate variability; (2) the sample size; (3) presence of autonomic neuropathy in the DGP patients. Mechanistically, EA at ST36 was reported to activate neurons in the nucleus tractus solitarius via somatic afferents and subsequently the dorsal motor nucleus of the vagus, resulting in an enhanced vagal efferent activity [43]. We speculated that via a similar neural pathway, TEA increased gastric vagal efferent activity, resulting in improved gastric pacemaking and motility activities and leading to improvement in gastroparesis symptoms.

The TEA method used in this study was completely noninvasive, easy‐to‐implement, self‐administrable and of low‐cost. In addition, it was reported to be safe without any serious side effects. These distinctive features make it attractive for the treatment of gastroparesis. However, further studies are needed to investigate its clinical efficacy and mechanisms of action.

There were several limitations in this pilot feasibility study. First, we did not perform the scintigraphic gastric emptying test. Second, the patients included in this study were refractory to medical therapies and may not be a representative sample for diabetic gastroparesis patients seen in the community. Therefore, our findings might not apply to all DGP patients. Third, this was a single‐center study with a relatively small sample size.

In conclusion, TEA at acupuncture points ST36 and PC6 with the special parameters is effective in treating major gastrointestinal symptoms in patients with diabetic gastroparesis. Further pivotal studies are needed to determine its clinical efficacy for treating diabetic gastroparesis.

## Author Contributions

Ying Zhu: data analysis and manuscript drafting. Irene Sarosiek: study design, data collection and analysis, manuscript revision. Yan Sun: data analysis. Jieyun Yin: study design, monitoring, data analysis. Thomas Abell: study design and manuscript revision. Borko Nojkov: data analysis and manuscript revision. Richard McCallum: study design, data collection, and manuscript revision. Jiande D.Z. Chen: study design and monitoring, data analysis, and manuscript drafting.

## Conflicts of Interest

Jieyun Yin is an employee of Transtimulation Research Inc. that made the TEA device used in this study.

## Data Availability

The data that support the findings of this study are available from the corresponding author upon reasonable request.
